# Room for improvement: One third of Lynch syndrome patients presenting for genetic testing in a highly specialised centre in Stockholm already have cancer

**DOI:** 10.1186/s13053-021-00171-4

**Published:** 2021-02-12

**Authors:** Sophie Walton Bernstedt, Jan Björk, Kaisa Fritzell, Allan D. Spigelman, Erik Björck, Ann-Sofie Backman

**Affiliations:** 1 Department of Medicine Solna, Karolinska Institutet, Stockholm, Sweden; 2 Division of Gastroenterology, Medical Unit Gastroenterology, Dermatovenereology and Rheumatology, Karolinska University Hospital, Stockholm, Sweden; 3 Patient flow Hereditary Cancer, Cancer Theme, Karolinska University Hospital, Stockholm, Sweden; 4 Department of Neurobiology, Care Sciences and Society, Division of Nursing, Karolinska Institutet, Stockholm, Sweden; 5 St Vincent’s Genetics Clinic, The Kinghorn Cancer Centre, Sydney, Australia; 6 St Vincent’s Clinical School, UNSW, Sydney, Australia; 7 Department of Molecular Medicine and Surgery, Karolinska Institutet, Stockholm, Sweden; 8 Clinical Genetics, Karolinska University Laboratory, Karolinska University Hospital, Stockholm, Sweden

**Keywords:** Lynch syndrome, Colorectal cancer, Genetic testing, Mismatch repair genes, Cancer prevention

## Abstract

**Background:**

Lynch syndrome is caused by germline mutations in the mismatch repair genes and is characterised by a familial accumulation of colorectal and other cancers. Earlier identification of Lynch syndrome patients enables surveillance and might reduce the risk of cancer. It is important to explore whether today’s clinical care discovers patients with Lynch syndrome suitable for surveillance in time. This study aimed to describe what led to a diagnosis of Lynch syndrome in the cohort referred to the Hereditary Gastrointestinal Cancer Unit, Karolinska University Hospital, Solna, Sweden for gastrointestinal surveillance.

**Methods:**

This was a descriptive study. Data from 1975 to 2018 were collected and compiled as a database. Age at diagnosis was calculated from the date when a pathogenic MMR gene mutation was confirmed, from the period June 1994–September 2018. Data were collected from patient protocols prospectively during patient consultations and medical records retrospectively. Criteria for inclusion were registration at the outpatient clinic and a confirmed mismatch repair gene mutation.

**Results:**

A total of 305 patients were eligible for inclusion. Three major reasons for diagnosis were identified: 1. Predictive testing of a previously known mutation in the family (62%, mean age 37), 2. A family history of Lynch associated tumours (9%, mean age 37), 3. A diagnosis of cancer (29%, mean age 51). The proportion diagnosed due to cancer has not changed over time.

**Conclusion:**

A high proportion of patients (29%) were identified with Lynch syndrome after they had been diagnosed with an associated cancer, which suggests that there is significant room for improvement in the diagnosis of patients with Lynch syndrome before cancer develops.

## Introduction

Lynch syndrome (LS) is caused by germline mutations in the mismatch repair genes and is characterised by an increased risk of developing colorectal cancer at a young age, slightly more frequent localisation of the tumour in the proximal colon and accumulation of colorectal and extracolonic cancers.

Early identification of those at high genetic risk of cancer might save lives because surveillance programmes would be indicated. Historically, in the absence of a known genetic cause, the primary diagnostic tool was an extensive family history. Vasen et al. [1] later introduced clinical criteria in 1991, known as the Amsterdam criteria 1, augmented by the Bethesda guidelines [2]. At present, the gold standard method to diagnose LS is by pedigree criteria and DNA sequencing. Due to the high lifetime risk of colorectal cancer (CRC) and endometrial cancer (EC), patients with LS are offered surveillance by means of colonoscopy and prophylactic hysterectomy. However, surveillance and prophylactic surgery only works if it is started in a timely manner given that the purpose is to prevent cancer. If genetic testing is offered in a limited way then a large proportion of patients at risk will remain undiagnosed until they present with cancer.

We wanted to explore to what extent clinical care discover patients with LS suitable for surveillance before the diagnosis of cancer. There are approximately 910 known individuals with Lynch syndrome in Sweden [3]. However, there are probably many undiagnosed cases as studies have indicated the prevalence of Lynch syndrome in the United States to be as high as 1/440 [4], which would correspond to a Swedish prevalence of 23,700 individuals. In Sweden 6000 cases of CRC [5] are diagnosed annually. Lynch syndrome is estimated to account for 2.2% of colorectal cancer cases in the US [6]. It is important to detect individuals with Lynch syndrome at an early age to offer surveillance to minimise the risk of developing cancer. The aim of the current study was to investigate causes what led to a diagnosis of LS and to identify changes over time among LS patients referred for endoscopic surveillance at Karolinska University Hospital, a highly specialised Swedish centre.

## Materials and methods

### Cohort description

Patients referred to the Hereditary GI Cancer Unit, Karolinska University Hospital, Sweden, for gastrointestinal surveillance, with a confirmed MMR gene mutation according to InSight Variant Interpretation Committee’s classification [7], or if the variant was unknown (class 3), reported to be pathogenic by the hospital’s genetics department after clinical evaluation based on family history were included. Approximately one third of Swedish patients with LS are followed at this clinic at the Karolinska University Hospital in Stockholm. This hospital has a catchment area that primarily includes Stockholm County, but acts as a second opinion hospital for north and mid-Sweden. The hospital has 1600 beds [8]. In 2018, Stockholm County had a population of around 2,3 million people [9] compared to Sweden as a whole which had a population of approximately 10 million [10].

### Data collection

Data from 1975 to 2018 were collected and compiled as a database (Table 1)*.* Age at diagnosis was calculated from the date when a pathogenic MMR gene mutation was confirmed, from the period June 1994–September 2018. Data were collected from patient protocols prospectively during patient consultations and medical records retrospectively.

**Table 1:** Demographic and clinical data on patients with Lynch syndrome in Stockholm County during the period June 1994–September 2018

Characteristic	N: 305 (%)
**Gender**	
Female	170 (56%)
Male	135 (44%)
**Age at diagnosis, mean (range)**	41 years (16–93)
**Age at inclusion, mean (range)**	45 years (16–92)
**Registered in Stockholm County**	282 (92%)
**Deceased**	13 (4%)
**Index visit**	235 (77%)
**Genotype**	
*MLH1*	142 (47%)
*MSH2*	83 (27%)
*MSH6*	43 (14%)
*PMS2*	30 (10%)
*EPCAM*	6 (2%)

### Variables

Possible reasons for investigation for LS were categorised as 1) predictive testing, 2) family history or 3) cancer, i.e. CRC, EC or skin tumour. Predictive testing was defined as when a person at risk is referred within the healthcare system to be tested for an MMR mutation that had previously been identified in the family. Family history was defined as when a patient was referred within the healthcare system to investigate whether they had Lynch syndrome or another hereditary cancer syndrome based on the family history of malignancies. Data regarding age, sex, cancer diagnoses (anatomical site and age at diagnosis and year of diagnosis) were collected. The genetic variants were verified for pathogenicity using the InSight Variant Interpretation Committee’s classification [7].

### Statistics

Descriptive statistics are presented as mean, range, proportions and percentages, comparison of data was made using Fischer’s exact test. Continuous data were evaluated to be normally distributed and were analysed using the Student’s t-test. Statistical significance was set at *P* < 0.05 with a confidence interval of 95%.

## Results

Of the 336 LS patients registered in the clinic; 305 were eligible for inclusion. Of the 305 patients 13 were found to be deceased, seven due to cancer-related causes. Five patients had missing information regarding cause of death and one patient had a cause of death not related to cancer. One patient was found to have mutations affecting multiple MMR genes (*MLH1* and *PMS2*). Patient demographic are shown in Table 1.

### Reasons for investigation leading to diagnosis of Lynch syndrome

**Fig. 1:**
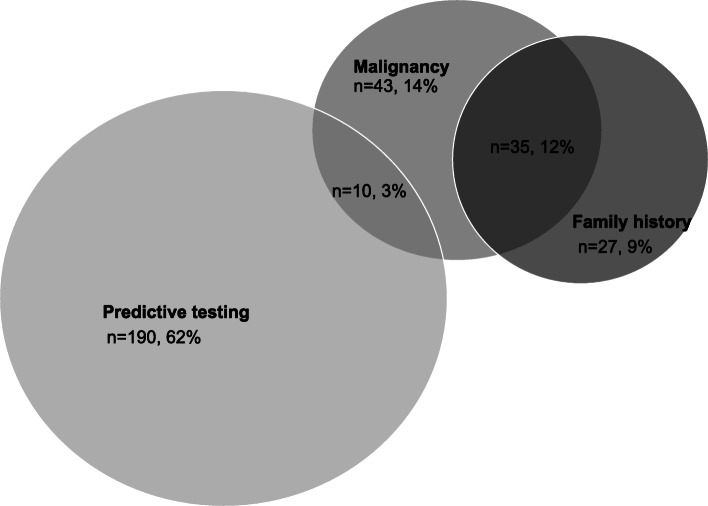
Reasons leading to a diagnosis of Lynch syndrome; Patients diagnosed by malignancies have been diagnosed due to colorectal cancer, endometrial cancer or skin tumour, data were collected on patients registered at the Hereditary Gastrointestinal Cancer Unit, Karolinska University Hospital, Solna, Sweden between the period June 1994–September 2018 (Figure created with Microsoft PowerPoint). 62% (*n* = 190) were diagnosed due to predictive testing. 3% (*n* = 10) were diagnosed due to a combination of predictive testing and malignancy. 14% (*n* = 43) were diagnosed due to malignancies. 12% (*n* = 35) were diagnosed due to a combination of malignancy and family history. 9% (*n* = 27) were diagnosed due to family history

Three major reasons were investigated 1. Pre-symptomatic predictive testing of previously known mutation in the family (62%, *n* = 190, mean age 37), 2. Family history of Lynch associated tumours (9%, *n* = 27, mean age 37), 3. Diagnosis of cancer (29%, *n* = 88, mean age 51) (Fig. 1.).

Of those with cancer almost half (51%, *n* = 45) had a family history consistent with LS or a familial known *MMR* gene mutation. The mean age at diagnosis of LS was 41 years (range 16–93 years). Those diagnosed due to any cancer were older than those diagnosed due to predictive testing and family history combined (mean 51 years, CI 48–53 years; mean 37 years, CI 35–39 years: *P* < 0.01). Those diagnosed with LS due to a diagnosis of CRC were diagnosed with CRC at a mean age of 44 years (range 24–75 years). There was no statistical significant difference in the cause for LS diagnosis observed between female and male patients regarding diagnosis due to cancer (28%, CI 22–36; 30%, CI 22–38: *P* = 0.80).

### Lynch syndrome – diagnosed due to cancer

Age at the diagnosis of cancer and the tumour spectrum that led to the diagnosis of LS is shown in Table 2. Patients (*n* = 88) diagnosed with LS due to a diagnosis of cancer were most frequently diagnosed due to CRC (*n* = 67, 76%) (Table 2). As a single diagnosis of cancer, CRC amounted to 72% (*n* = 63). Other single diagnoses of cancer that led to a diagnosis of LS were EC (*n* = 16, 18%), skin tumours (*n* = 3, 3%) and ovarian cancer (*n* = 1, 1%). Five (6%) patients had multiple cancer diagnoses that led to a diagnosis of LS.

**Table 2:** Tumour spectrum and age at diagnosis of each tumour leading to a diagnosis of Lynch syndrome

Patients	N:88 (%)	Age at diagnosis mean (range)
**Colorectal cancer**	67 (76%)	(44, 24–75)
**Extracolonic cancer**	25 (28%)	(49, 24–65)
**Gynaecological cancer**	21 (24%)	(49, 38–65)
**Skin tumour**	4 (5%)	(45, 24–58)
**Total number of tumours**^a^	117	

^a^Five (6%) patients had multiple cancer diagnoses that led to a diagnosis of Lynch syndrome

Most patients (78%, *n* = 52) with a diagnosis of CRC that led to a diagnosis of Lynch syndrome were diagnosed with CRC due to anaemia and/or symptoms. Four patients were diagnosed in the context of a general endoscopic CRC screening programme or due to endoscopic surveillance motivated by a family history of CRC. Four patients were diagnosed due to other CRC surveillance such as a general CRC surveillance programme or due to a family history of CRC. Eleven patients had missing information regarding cause of CRC diagnosis.

Those patients who were diagnosed with gynaecological cancer, presented most often with symptoms such as uterine bleeding and abdominal discomfort (71%, *n* = 15). Other causes leading to a diagnosis of gynaecological cancer were follow-up examination due to CRC, follow-up due to a family history of cancer and follow-up due to miscarriage. Of those patients diagnosed due to skin tumours, three were suspected of having LS due to the presence of sebaceous tumours and one patient had dysplastic nevi.

### Reasons for diagnosis of Lynch syndrome in patients at or over 60 years of age

**Fig. 2:**
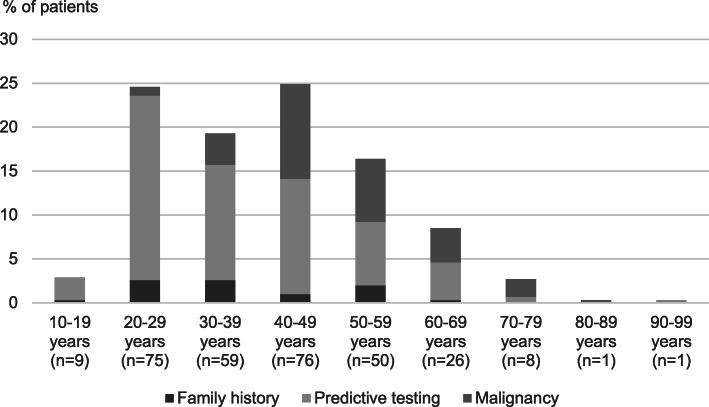
Reason for diagnosis of Lynch syndrome in relation to age, *n* = 305; In the age range 10–19 years, 0.3% (*n* = 1) were diagnosed due to family history and 2.6% (*n* = 8) due to predictive testing. In the age range 20–29 years 2.6% (*n* = 8) were diagnosed due to family history, 21% (*n* = 64) were diagnosed due to predictive testing and 1% (*n* = 3) were diagnosed due to malignancy. In the age range 30–39 years, 2.6% (*n* = 8) were diagnosed due to family history, 13.1% (*n* = 40) were diagnosed due to predictive testing and 3.6% (*n* = 11) were diagnosed due to malignancy. In the age range 40–49 years, 1% (*n* = 3) were diagnosed due to family history, 13.1% (*n* = 40) were diagnosed due to predictive testing and 10.8% (*n* = 33) were diagnosed due to malignancy. In the age range 50–59 years, 2% (*n* = 6) were diagnosed due to family history, 7.2% (*n* = 22) were diagnosed due to predictive testing and 7.2% (*n* = 22) were diagnosed due to malignancy. In the age range 60–69 years, 0.3% (*n* = 1) were diagnosed due to family history, 4.3% (*n* = 13) were diagnosed due to predictive testing and 3.9% (*n* = 12) were diagnosed due to malignancy. In the age range 70–79 years, 0.7% (*n* = 2) were diagnosed due to predictive testing and 2% (*n* = 6) were diagnosed due to malignancy. In the age range 80–89 years, 0.3% (*n* = 1) were diagnosed due to malignancy. In the age range 90–99 years, 0.3% (*n* = 1) were diagnosed due to malignancy

Reasons for diagnosis of Lynch syndrome in relation to age, *n* = 305, are described in Fig. 2. LS was diagnosed in 36 patients at or over 60 years of age. Among these, the majority (*n* = 20, 56%) had received their diagnosis in relation to a diagnosis of cancer. A third (*n* = 12, 33%) explicitly requested a genetic investigation. Other reasons that warranted genetic investigation included making genetic screening available for relatives (*n* = 8, 22%), offering preventive measures such as endoscopies, hysterectomy and adequate follow-up after cancer (*n* = 12, 33%) and research purposes (*n* = 4, 11%).

### Reasons for investigation leading to diagnosis of Lynch syndrome - time trend

**Fig. 3:**
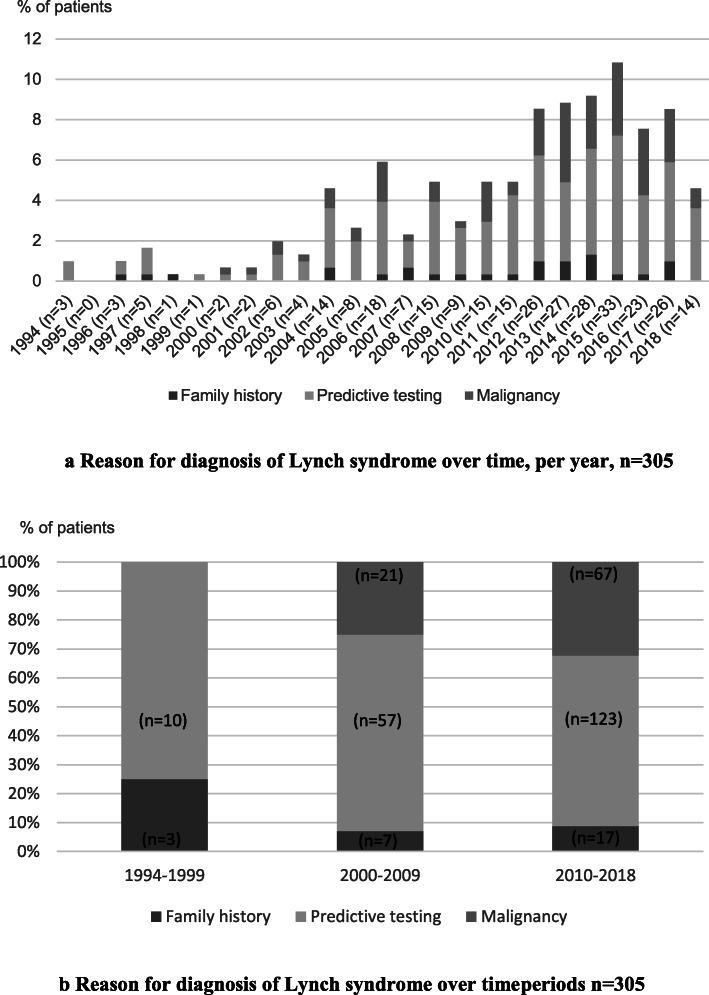
**a** Reason for diagnosis of Lynch syndrome over time, per year, *n* = 305; Data were collected on patients registered at the Hereditary Gastrointestinal Cancer Unit, Karolinska University Hospital, Solna, Sweden between the period June 1994–September 2018 (Figure created with Microsoft Excel). In 1994, 1% (*n* = 3) patients were diagnosed due to predictive testing. In 1995, 0% (*n* = 0) patients were diagnosed. In 1996, 0.3% (*n* = 1) were diagnosed due to family history and 0.7% (*n* = 2) due to predictive testing. In 1997, 0.3% (*n* = 1) were diagnosed due to family history and 1.3% (*n* = 4) were diagnosed due to predictive testing. In 1998, 0.3% (*n* = 1) were diagnosed due to family history. In 1999, 0.3% (*n* = 1) were diagnosed due to predictive testing. In 2000, 0.3% (*n* = 1) were diagnosed due to predictive testing and 0.3% (*n* = 1) were diagnosed due to malignancy. In 2001, 0.3% (*n* = 1) were diagnosed due to predictive testing and 0.3% (*n* = 1) were diagnosed due to malignancy. In 2002, 1.3% (*n* = 4) were diagnosed due to predictive testing and 0.7% (*n* = 2) due to malignancy. In 2003, 1% (*n* = 3) were diagnosed due to predictive testing and 0.3% (*n* = 1) were diagnosed due to malignancy. In 2004, 0.7% (*n* = 2) were diagnosed due to family history, 3% (*n* = 9) were diagnosed due to predictive testing and 1% (*n* = 3) were diagnosed due to malignancy. In 2005, 2% (*n* = 6) were diagnosed due to predictive testing and 0.7% (*n* = 2) were diagnosed due to malignancy. In 2006, 0.3% (*n* = 1) were diagnosed due to family history, 3.6% (*n* = 11) due to predictive testing and 2% (*n* = 6) due to malignancy. In 2007, 0.7% (*n* = 2) were diagnosed due to family history, 1.3% (*n* = 4) were diagnosed due to predictive testing and 0.3% (*n* = 1) were diagnosed due to malignancy. In 2008, 0.3% (*n* = 1) were diagnosed due to family history, 3.6% (*n* = 11) were diagnosed due to predictive testing and 1% (*n* = 3) were diagnosed due to malignancy. In 2009, 0.3% (*n* = 1) were diagnosed due to family history, 2.3% (*n* = 7) were diagnosed due to predictive testing and 0.3% (*n* = 1) were diagnosed due to malignancy. In 2010, 0.3% (*n* = 1) were diagnosed due to family history, 2.6% (*n* = 8) were diagnosed due to predictive testing and 2% (*n* = 6) were diagnosed due to malignancy. In 2011, 0.3% (*n* = 1) were diagnosed due to family history, 3.9% (*n* = 12) were diagnosed due to predictive testing and 0.7% (*n* = 2) were diagnosed due to malignancy. In 2012, 1% (*n* = 3) were diagnosed due to family history, 5.2% (*n* = 16) were diagnosed due to predictive testing and 2.3% (*n* = 7) were diagnosed due to malignancy. In 2013, 1% (*n* = 3) were diagnosed due to family history, 3.9% (*n* = 12) were diagnosed due to predictive testing and 3.9% (*n* = 12) were diagnosed due to malignancy. In 2014, 1.3% (*n* = 4) were diagnosed due to family history, 5.2% (*n* = 16) were diagnosed due to predictive testing and 2.6% (*n* = 8) were diagnosed due to malignancy. In 2015, 0.3% (*n* = 1) were diagnosed due to family history, 6.9% (*n* = 21) were diagnosed due to predictive testing and 3.6% (*n* = 11) were diagnosed due to malignancy. In 2016, 0.3% (*n* = 1) were diagnosed due to family history, 3.9% (*n* = 12) were diagnosed due to predictive testing and 3.3% (*n* = 10) were diagnosed due to malignancy. In 2017, 1% (*n* = 3) were diagnosed due to family history, 4.9% (*n* = 15) were diagnosed due to predictive testing and 2.6% (*n* = 8) were diagnosed due to malignancy. In 2018, 3.6% (*n* = 11) were diagnosed due to predictive testing and 1% (*n* = 3) were diagnosed due to malignancy. **b.** Reason for diagnosis of Lynch syndrome over timeperiods, *n* = 305; Data were collected on patients registered at the Hereditary Gastrointestinal Cancer Unit, Karolinska University Hospital, Solna, Sweden between the period June 1994–September 2018 (Figure created with Microsoft Excel). In 1994–1999, 77% (*n* = 10/13) patients were diagnosed due to family history and 23% (*n* = 3/13) were diagnosed due to predictive testing. In 2000–2009, 25% (*n* = 21/85) were diagnosed due to malignancy, 67% (*n* = 57/85) were diagnosed due to predictive testing and 8% (*n* = 7/85) were diagnosed due to family history. In 2010–2018, 32% (*n* = 67/207) were diagnosed due to malignancy, 60% (*n* = 123/207) were diagnosed due to predictive testing and 8% (*n* = 17/207) were diagnosed due to family history

Figure 3a shows the number of cases diagnosed with Lynch syndrome over time year by year in relation to the reason for diagnosis. Those who received their diagnosis 2010–2018 amounted to 68% (*n* = 207/305) of the cohort and of these patients 32% (*n* = 67/207) received their diagnosis due to genetic testing initiated because of a diagnosis of cancer (Fig. 3b). Cancer was found to be a cause for initiation of an investigation leading to a diagnosis in almost one third of patients between the years 2010–2018, mostly due to CRC. Between the periods 2000–2009 and 2010–2018, there has been no statistically significant change in the proportion diagnosed due to cancer (25%, CI 16–34%; 32%, CI 26–39%: *P =* 0.21).

## Discussion

In this cohort, three major causes leading to a diagnosis of Lynch syndrome were investigated: predictive testing, cancer and family history. The most common cause for diagnosis was predictive testing. Approximately a third of patients received their diagnosis due to a previous diagnosis of cancer. It has been suggested [11] that up to 77% of relatives of patients with CRC or endometrial cancer diagnosed with Lynch syndrome are unaffected at the time of genetic testing. Win et al. [12] have also shown that 7% of patients with LS may have more than one cancer at the time of diagnosis. The most common cancer diagnosis leading to a diagnosis of LS in the current study was CRC, followed by gynaecological and skin tumours. Those diagnosed due to CRC were diagnosed at a mean age of 44, a result similar to previous studies [13]. Most patients (69%) received their diagnosis at 20–49 years, but patients up to 93 years in the studied cohort were investigated and found to have LS. Investigating MMR status in patients with CRC up to 70 years of age is beneficial in order to increase the detection of LS since these patients may not meet previous criteria for genetic testing [14]. Our findings support this notion and show that testing for LS in even older patients may be beneficial.

The number of patients diagnosed with LS increased over time but over the last two decades the reasons for diagnosis have not changed. More patients are thus now identified as having LS. Patients are still not identified in time to prevent cancer development, suggesting a large number of cases remaining undiagnosed. Mittendorf et al. [15] found that as many as 64% were diagnosed with a LS related malignancy prior to their diagnosis of Lynch syndrome. Mittendorf et al. [15] also found that prior to 2009, 11% of patients received a diagnosis of Lynch syndrome within 1 year of a related cancer compared to after 2009 when 83% received a diagnosis. The increasing number of diagnosed patients could imply increasing awareness among colorectal surgeons and oncologists who now initiate genetic investigations more often in the case of cancer in a young person. It has, on the other hand, been demonstrated that LS is under-recognised, even when patients have clear criteria unrelated to family history. Singh et al. [16] found that as few as 7% of patients with CRC who meet the Bethesda guidelines were offered genetic counselling. Another study [17] showed that 11% of patients who met the Bethesda guidelines and 25% of individuals who met the Amsterdam II criteria were screened for Lynch syndrome. Adelson et al. [18] have also demonstrated that only 22% of patients (22/97) with CRC who the Bethesda guidelines applied to underwent further investigation, and that one third of physicians underestimate the penetrance of Lynch syndrome [19] which might be improved by education and structural changes.

After a diagnosis of LS, the clinical routine in Sweden is to ask patients to inform their relatives about the option of presymptomatic predictive testing. This strategy has not been successful [20] whereas when healthcare professionals directly contact relatives the testing rate almost doubles [21]. It has been suggested that relatives may not comprehend information provided and that when adequately informed, rate of testing will increase [22, 23]. Up to 66% of relatives preferred to receive information from the hospital rather than from family members [24].

Strengths of the study include the low risk of interobserver bias, as all patient data were collected in a standardised manner in the form of protocols by just a few authors. Limitations include the relatively small sample size studied. Patients included in the study were found to have migrated from other counties and countries, accounting for some missing data. Changes in the electronic medical record systems over time also limited access to previous information.

## Conclusion

We conclude that a high proportion of patients were initially identified with Lynch syndrome after they had been diagnosed with an associated cancer and that this proportion has not changed over recent years. This finding supports the importance of improving the awareness about these high risk genes among clinicians and health policy makers to increase the identification of patients with Lynch syndrome and thereby facilitate the prevention of cancer.

## Data Availability

All raw data are securely stored within the Hereditary Gastrointestinal Cancer Unit, Karolinska University Hospital.
